# Adipose-Derived Mesenchymal Stem Cells (AD-MSCs) against Ultraviolet (UV) Radiation Effects and the Skin Photoaging

**DOI:** 10.3390/biomedicines9050532

**Published:** 2021-05-11

**Authors:** Pietro Gentile, Simone Garcovich

**Affiliations:** 1 Department of Surgical Science, Plastic and Reconstructive Surgery, Medical School, “Tor Vergata” University, 00133 Rome, Italy; 2 Scientific Director of Academy of International Regenerative Medicine & Surgery Societies (AIRMESS), 1201 Geneva, Switzerland; 3 Institute of Dermatology, F. Policlinico Gemelli IRCSS, Università Cattolica del Sacro Cuore, 00168 Rome, Italy; simgarko@yahoo.it

**Keywords:** adipose-derived mesenchymal stem cells (AD-MSCs), autologous AD-MSCs, autologous fat transplants, fat grafting, skin photoaging, stem cell therapy, regenerative plastic surgery

## Abstract

The skin is a natural barrier against the ultraviolet (UV) radiation of sunlight. The long-term and/or repetitive exposure to the sunlight and related UV radiation may change the skin structure, decreasing collagen production, promoting premature skin aging, which is termed “photoaging”. The signs of photoaging include wrinkle formation, mottled pigmentation, and/or cancerous changes. For many years, adipose-derived mesenchymal stem cells (AD-MSCs) and fat grafting (F-GRF) have been used to combat photoaging signs, wrinkles, loss of elasticity, and face soft tissue defects. Several studies have analyzed in vitro actions of AD-MSCs against photoaging’s effects, thanks to their migratory activity, paracrine actions, and related in vivo–ex vivo outcomes. In fact, AD-MSCs act against skin photoaging in vitro via activation of dermal fibroblast proliferation, antioxidant effect, and matrix metalloproteinases (MMPs) reduction. In vivo and ex vivo outcomes regard the local injection of AD-MSCs, F-GRF, and/or enriched-F-GRF with AD-MSCs directly in the wrinkles and the face’s soft tissue defects. This concise review summarizes the most recent in vitro, in vivo and ex vivo outcomes and developments on the effects of AD-MSCs and F-GRF against photoaging.

## 1. Introduction

The skin is a natural barrier against the ultraviolet (UV) radiation of sunlight [1]. The acute exposure to UV radiation leads to sunburn and local tissue damage, while long-term and/or repetitive exposure may change the skin structure, decreasing collagen production and promoting premature skin aging, which is termed “photoaging” [2]. Therefore, the UV exposure damages the skin’s stratum corneum, reduces its protective capacity, and leads to water loss. Large doses of UV radiation may enter deeply into the dermis, contributing to collagen degeneration as well as wrinkle formation [3]. Additionally, hereditary factors may facilitate the onset of photoaging’s signs, represented by wrinkle formation, major roughness, loss of elasticity, soft tissue defects with volume loss, and aging.

UV is mainly composed of UV-A (wavelengths ranging from 320 to 420 nm) and UV-B (wavelengths ranging from 275 to 320 nm), altering specific parts of the skin tissue and leading to diverse consequences. In fact, UV-B radiation mainly affects the epidermis; when it is repetitive, it causes redness and a reduction of the skin’s elasticity, and it promotes wrinkle formation. UV-A radiation may penetrate deeply into the dermis and damage DNA, causing photoaging. In vitro studies have shown that the skin’s photoaging is characterized both by minimal epidermal changes and by major dermal alterations, indicating that UV-A plays a pivotal role in photoaging [4]. Disordered collagen fibrils and a mass of abnormal elastic material in the superficial and mid dermis have been identified. The negative in vivo effect of UV radiation is in fact on collagen production, leading to fine or coarse wrinkle development, an increase in skin roughness and fragility, mottled pigmentation, loss of elasticity and volume, and precancerous and cancerous changes [4].

The number of investigations evaluating the efficacy of autologous adipose-derived mesenchymal stem cells (AD-MSCs) contained in the stromal vascular fraction (SVF) of fat grafting (F-GRF) in the face’s soft-tissue defects and signs of aging has exponentially increased during the last twenty years (2000–2020).

Autologous F-GRF is an interesting procedure in regenerative plastic surgery eagerly used in a growing number of indications, from skin rejuvenation and lipofilling to wound treatment [5]. Most of fat grafts’ regenerative capacity is attributed to AD-MSCs, suspended in a fatty tissue cellular matrix—SVF [6]. It consists of a mixture of endothelial, smooth muscle cells, pericytes, and leukocytes [7]. The percentage of AD-MSCs in SVF, varies depending on the isolation method, but it is greater than in classic fat graft [8]. New approaches to fat processing have emerged. Micro-fat and its derivate—nano-fat—became promising methods of lipofilling, even in superficial skin layers [8,9]. Several techniques based on centrifugation, emulsification, and filtration procedures have been described to obtain nano-fat and micro-fat [10].

SVF and AD-MSCs were identified and described by the International Federation for Adipose Therapeutics and Science (IFATS) in combination with the International Society for Cellular Therapy (ISCT), as recently analyzed [10]. As per their description, cells needed a viability of ≥70% and ≥90% for SVF and AD-MSCs, respectively. The SVF’s identity, phenotype, and functional characteristics were considered closely related to the adipose microenvironment and identified through a typical marker profile. In detail, the SVF immunophenotype showed the following typical marker profile for stromal cells: CD44, CD73, CD13, CD90, and CD29 positive (≥40%) and CD34 positive (≥20%) but CD31 (≤20%) and CD45 negative (≤50%). In contrast, AD-MSCs were positive for CD29, CD13, CD44, CD90, CD73, and CD105 (>80%) but negative for CD31, CD45, and CD235a (≤2%) [10]. In addition, AD-MSCs are expected to have the potential to differentiate into the osteogenic, chondrogenic, and adipogenic lineage. Generally, each mL of human adipose tissue offers 300,000 cells, of which 1–3% are represented by ASCs (3000–9000/mL) [10,11].

SVF and AD-MSCs improve adipogenesis, vascularization, and growth factor production; hence, some have tried using them in scar treatment [12,13,14].

The aim of regenerative strategies against skin photoaging must be the development of new autologous-biotechnologies that involve AD-MSCs and SVF by ex vivo and in vitro culture or by in vivo regeneration and bio-stimulation.

In this concise review, data from recent investigations reporting the use of AD-MSCs, SVF, and F-GRF in photoaging and face soft tissue defects treatment are used to evaluate such interventions’ efficacy, while related biomolecular signaling pathways were analyzed.

## 2. UV-Caused Skin Photoaging Biomolecular Signaling Pathway

Two main signaling pathways in the biomolecular process of UV-caused skin photoaging have been identified: the MAPK signaling pathway and the TGF-β/Smad signaling pathway.

In the first signaling pathway (MAPK), UV irradiation may activate cell surface receptors for interleukin (IL)-1, epidermal growth factor (EGF), and tumor necrosis factor (TNF)-α, which leads to the phosphorylation of the mitogen-activated protein kinases (MAPKs), including c-Jun amino terminal kinase (JNK) and P38; this kinase activation will further induce the transcription of the activator protein 1 (AP-1) and nuclear factor kappa B (NF-κB) [15,16]. The activation of AP-1 and NF-κB habitually affects the expression of matrix metalloproteinases (MMPs), such as MMP-1 and MMP-3. Excessive MMPs are considered as a critical factor accountable for the pathology of skin photoaging diseases, such as collagen degradation and wrinkle formation [15,16].

The second signaling pathway (TGF-β/Smad) involves the modulation of transforming growth factor-β (TGF-β) and Smad. The TGF-β/Smad signaling transduction regulated by UV is a key factor that leads to skin photoaging [17,18]. Under common conditions, TGF-β initially binds to TGF-β receptor type II (TβRII), a specific cell-surface receptor, and further activates the intrinsic kinase activity of TGF-β receptor type I (TβRI). Afterward, the transcription factors Smad2 and Smad3 will be successively phosphorylated and combined with Smad4, which will regulate the transcription of specific genes, including the biosynthesis of procollagen [19]. UV irradiation of human skin impairs TGF-β/Smad signaling by downregulating TβRII expression and protein synthesis. In human skin fibroblasts, this process is mediated by transcriptional repression, which contributes to considerable reduction of TGF-β responsiveness. Then, the inhibition of TGF-β prevents activation of Smad2/3, thereby production [18]. Reduced procollagen expression is a key feature of the photoaged skin.

## 3. The Protective Role of AD-MSCs in Skin Photoaging: In Vitro and In Vivo Analysis

Several findings have shown that F-GRF and related AD-MSCs/SVF are effective in treating soft tissue defects and wrinkles [11,20,21,22,23], and previous studies have emphasized their application in wound healing (WH), scars treatment (ST), and also in the treatment of radiotherapy damage [24]. Thanks to the therapeutic effects of AD-MSCs and SVF in WH and ST, great attention has been paid to utilizing AD-MSCs in the treatment of photoaging damage. The local injection of autologous AD-MSCs and/or of F-GRF in the photoaged skin area promotes new collagen synthesis and epidermal thickening and reduces visible wrinkling [25,26].

An autologous AD-MSCs use as an alternative therapy for repair of photodamaged tissues promises to become predominant in the tissue regeneration field.

Son et al. [16] analyzed the effects of AD-MSCs in UV-irradiated human dermal fibroblasts (HDFs) for therapeutic potential in skin wrinkling. UV irradiation, a model that naturally mimics skin wrinkle formation, is known to increase matrix metalloproteinase-1 (MMP-1), making MMP-1 a target for skin photoaging. Son et al. [16] identified that AD-MSCs reduce MMP-1 level in UV-irradiated HDFs and increase type 1 procollagen in HDFs. A dose-dependent increase in type 1 procollagen was confirmed by AD-MSC-conditioned medium (AD-MSCs-CM). These findings showed the effects of AD-MSCs on the induction of MMP-1 in UV-radiated HDFs and the expression of collagen in HDFs, providing evidence of the relationship between MMP-1 and procollagen production for protection against wrinkle formation.

In in vitro models, the protective role of AD-MSCs via AD-MSCs-CM treatment in UVB-radiated human keratinocyte cells and HDFs has been investigated. The keratinocyte cells and dermal fibroblasts, which maintain the structural integrity of skin tissue, are considered as basic barriers against UV radiation. Recent studies have shown that AD-MSCs-CM could stimulate the synthesis of collagen and procollagen, increase dermal thickness, enhance the migration of HDFs, inhibit MMP production, and promote WH [25]. AD-MSCs display multi-lineage developmental plasticity and secrete various factors such as vascular endothelial growth factor (VEGF), hepatocyte growth factor (HGF), interleukin-6 (IL-6), insulin-like growth factor (IGF), and pigment epithelium-derived factor (PEDF). These factors can activate the proliferation of HDFs and regulate collagen synthesis, thus protecting the neighboring cells from photoaging and improving the appearance of wrinkles [27]. Moreover, AD-MSCs-CM has been reported to promote TGF-β, another crucial cytokine that is involved in HDF proliferation and collagen synthesis. It is well established that TGF-β can modulate cell proliferation, differentiation, and extracellular matrix (ECM) production; therefore, the capacity of AD-MSCs-CM to promote TGF-β production and keep TGF-β from the disturbance of UV irradiation is quite critical for its photo-protective effects [28]. In UV-irradiated photoaged skin tissue, there is distinct activation of MAPK signaling and suppression of TGF-β/Smad signaling, which leads to the perturbation of procollagen synthesis [29]. In recent in vitro studies, it has been reported that AD-MSCs-CM can effectively block the MAPK signaling pathway and inhibit related signaling transducers, such as AP-1 and NF-κB [25]. The downregulation of the MAPK signaling pathway will decrease proinflammatory proteins, such as IL-6, to alleviate UV-induced stress at an early stage. In addition, AD-MSCs-CM can efficiently promote antioxidant response element (ARE), which increases TGF-β expression and collagen synthesis [25,28]. As reported, AD-MSCs-CM could inactivate the MAPK signaling pathway, block the expression of AP-1 signaling transducers, and inhibit the synthesis of proinflammatory factors such as IL-6; consequently, excessive induction of MMPs in skin can be efficiently downregulated [25,28]. Notably, both AD-MSCs and fibroblasts can significantly promote collagen synthesis, while fibroblasts are reported to increase the expression of MMPs, especially MMP-13, which is quite opposite to the effect of AD-MSCs. This kind of difference may potentially explain the deeper way in which AD-MSCs wield their photo-protective effects, although many barriers remain to be conquered [26].

In pre-clinical experiments, Jeong et al. [26] focused on the effects of AD-MSCs and HDFs in reducing wrinkles on photoaged skin in nude mice. Their results showed that the AD-MSCs-treated group reported more remarkable improvement of wrinkles, with higher collagen density.

In in vivo studies and related clinical trials, Luiz Charles-de-Sá et al. [30] have injected AD-MSCs expanded in vitro to the facial skin of patients after local operation. The analysis of elastic matrix components displayed a thorough regeneration of elastic oxytalan and elaunin fibers in the sub-epidermal region and the reconstruction of normal elastin fiber network in the dermis, indicating that AD-MSCs function as an appropriate access to structural restoration of photo-damaged skin [30].

The in vivo efficacy of AD-MSCs, SVF, and F-GRF was primarily evaluated by a reduction in healing’ time and scars area during WH and by an improvement of soft tissue volume maintenance and skin quality during ST and wrinkle treatment (WT). Secondarily, efficacy was demonstrated by satisfaction of patients reported in surveys, and by observation of texture changes of treated areas when comparing photographs and histological analysis taken before and after the treatment. Given that various testing methods were used in the included studies, only the most widely used methods would be set as endpoints for all pooled studies. All side effects, including local injection pain and increasing sensitivity in the treated area, were analyzed. Many of the analyzed studies showed an improvement in soft tissue volume maintenance and skin quality and a reduction of scarred area; healing time during ST and WH confirmed the safety and efficacy. Compared with the control group and baseline, a few pooled patients reported adverse effects, including mild pain on harvesting sites (during the procedures and/or after 48 to 72 h, which would resolve spontaneously) and skin sensitivity on injection sites on the fourth- or fifth-day post-operation [10,11,20]. At the same time, it has been possible to highlight that there is a lack of a standardized and widely shared protocol for the isolation methods/preparation method of AD-MSCs and SVF, as well as the lack of standardized evaluation procedures.

## 4. The Protective Role of AD-MSCs in Oxidative Stress: In Vitro and In Vivo Analysis

In addition to the effects on ECM, UV also generates intracellular reactive oxygen species (ROS), a crucial factor leading to damage in photoaged skin tissue [31]. Normally, the intracellular ROS levels are controlled within normal range by the cellular defense system, while UV exposure disrupts the activities of defensive enzymes and increases the production of intracellular ROS. An overproduction of ROS will disturb the HDFs and induce the activation of MMP, which breaks the balance between synthesis and degradation of ECM, leading to excessive degradation of interstitial collagen [3,32]. Moreover, massive ROS damages the membrane lipids and releases arachidonic acid, which can be converted into prostaglandins to attract inflammatory cells to the injured area, resulting in further damage. The cellular defense system against ROS is composed of anti-oxidative defense enzymes and small molecular antioxidants, such as catalase, glutathione peroxidase (GPx), and superoxide dismutase (SOD), which can scavenge ROS and keep the affected cells from oxidative stress. Thus, we can reasonably summarize two possible methods to reduce intracellular ROS, that is, to directly alter ROS production or to activate the antioxidant defense system [33]. The MSCs possess excellent ROS-scavenging capacity and remarkably promote the cellular antioxidant defense system, facilitating resistance against UV, as reported in an article on bone marrow MSCs [34]. The antioxidant effect of MSCs can be demonstrated through increased activities of SOD and GPx in HDFs because SOD and GPx can effectively promote the survival rates of HDFs and play crucial roles in maintenance of HDF morphology [34]. Additionally, the coordinate secretion of soluble factors that protect HDFs from oxidative stress are also involved in the antioxidant action of MSCs, resulting in thickening epidermis, smoother skin, and less visible wrinkles.

Both morphological alterations and protein analysis reveal that AD-MSCs and AD-MSCs-CM effectively protect HDFs from oxidative stress induced by UV irradiation.

Li et al. [25] reported the anti-oxidative capacity of the AD-MSCs via upregulating ARE, such as phase II gene heme oxygenase-1 (HO-1), and increasing the expression of collagen synthesis enhancer gene TGF-β. Kim et al. [35] reported the capacity of the AD-MSCs to enhance the proliferation and migration of HDFs through paracrine effects, resulting in acceleration of wound healing [35].

**Figure 1:**
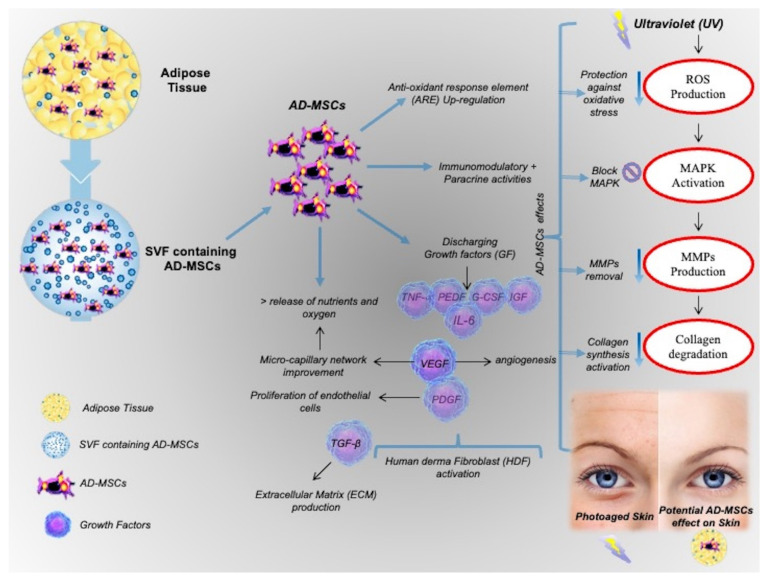
The protective role of AD-MSCs.

Furthermore, in AD-MSCs-CM, many kinds of antioxidant cytokines, such as SOD, IGF, PEDF, HGF, and IL-6, are successively detected by proteomic analysis in conjunction with other medical assays [36,37]. To be specific, IGF is reported to efficiently protect fibroblasts from free radicals, while PEDF proves to be an anti-angiogenic factor with anti-oxidative capacity. HGF and IL-6 can significantly reduce oxidative stress induced by glutathione depletion and by hydrogen peroxide, respectively [36,37]. In pre-clinical models, AD-MSCs-CM can also resist oxidation by promoting collagen regeneration and accelerating wound healing, and the activities of both SOD and GPx are notably increased in dermal fibroblasts. The protective role of AD-MSCs is shown in Figure 1.

## 5. The Effect of Exosomes on Skin Photoaging

The topical application of exosomes secreted by MSCs (MSC-Exos) on the skin is a very new and interesting topic in the medical field. Zhang et al. [38] investigated whether marine sponge Haliclona sp. spicules (SHSs) could effectively enhance the skin delivery of human umbilical cord-derived MSC-Exos (hucMSC-Exos), and further evaluated the topical application of hucMSC-Exos combined with SHSs in rejuvenating photoaged mouse skin. The combined use of hucMSC-Exos and SHSs showed in vitro significant anti-photoaging effects in mice, including reducing microwrinkles, alleviating histopathological changes, and promoting the expression of extracellular matrix constituents, whereas hucMSC-Exos alone produced considerably weaker effects. Zhang et al. [38] concluded that the combination of MSC-Exos and SHSs may be of much use in the treatment of photoaging. Liang et al. [39] reported the antiaging effects of AD-MSC-derived exosomes on photoaged skin. Human AD-MSCs were isolated from the fat of healthy women and cultured in vitro. Then, exosomes were extracted from the cultured AD-MSCs, purified by ultracentrifugation, and verified by examination of cell morphology using transmission electron microscopy and the identification of specific biomarkers. The photoaged skin model was created by subjecting Sprague-Dawley rats to UVB radiation. Exosomes were injected into the photoaged skin in a single therapeutic dose. The thickness of the epidermis and dermis was observed by HE staining. The relative mRNA expression of type I collagen, type III collagen, and matrix metalloproteinases (MMP-1 and MMP-3) was determined by real-time PCR. In the rat model of photoaged skin, the injected exosomes markedly decreased the epidermal thickness and increased the dermal thickness of the photoaged skin 7 days after treatment. Moreover, the proportion of the stratum corneum of the epidermis was decreased. Furthermore, real-time PCR showed that the mRNA expression of type I collagen was increased and that of type III collagen, MMP-1, and MMP-3 was decreased. Liang et al. [39] demonstrated that AD-MSC-derived exosome treatment could significantly improve skin photodamage and that AD-MSCs-derived exosomes may be a potential agent for photoaged skin treatment.

## 6. Conclusions

AD-MSCs protective roles and their restorative action in skin photoaging induced by UV radiation, through the activation of HDF proliferation, synthesis of collagen, protection against oxidative stress, and the reduction of MMPs, were analyzed. The protective role of AD-MSCs against oxidative stress is based on promoting the proliferation of HDFs, which, in turn, inhibit ROS production via secretion of various cytokines.

In conclusion, the results analyzed showed that AD-MSCs and also AD-MSCs-derived exosomes might be potential cosmo-therapeutic tools for addressing the problem of photoaging.
